# Anisodamine for the prevention of contrast-induced nephropathy in patients with acute coronary syndrome: a pilot systematic review and meta-analysis of randomized controlled trials

**DOI:** 10.1097/MS9.0000000000002181

**Published:** 2024-05-21

**Authors:** Hritvik Jain, Ramez M. Odat, Jyoti Jain, Debankur Dey, Ayham Mohammad Hussein, Mohammed Dheyaa Marsool Marsool, Haania Shahbaz, Aniket Mathur, Himani Yadav, Siddhant Passey, Rukesh Yadav

**Affiliations:** a Department of Internal Medicine, All India Institute of Medical Sciences (AIIMS), Jodhpur; b Medical College Kolkata, Kolkata, West Bengal; c Department of Internal Medicine, Jhalawar Hospital and Medical College, Jhalawar, Rajasthan, India; d Faculty of Medicine, Jordan University of Science and Technology, Irbid; e Faculty of Medicine, Al-Balqa’ Applied University, Salt, Jordan; f Department of Internal Medicine, Al-Kindy College of Medicine, University of Baghdad, Baghdad, Iraq; g Department of Internal Medicine, Dow University of Health Sciences, Karachi, Pakistan; h Department of Internal Medicine, University of Connecticut Health Center, CT, USA; i Department of Internal Medicine, Maharajgunj Medical Campus, Institute of Medicine, Tribhuvan University, Kathmandu, Nepal

**Keywords:** acute myocardial infarction, creatinine, anisodamine, contrast-induced nephropathy, percutaneous coronary intervention

## Abstract

**Introduction:**

Contrast-induced nephropathy (CIN) is a common post-procedural complication of percutaneous coronary intervention for acute myocardial infarction (AMI). Anisodamine hydrobromide is an alkaloid that has demonstrated efficacy in improving microcirculation. This meta-analysis aims to evaluate the reno-protective effects of Anisodamine in patients undergoing percutaneous coronary intervention (PCI) for AMI.

**Methods:**

PubMed, Embase, Cochrane Library, Scopus, and clinicaltrials.gov were searched from inception to January 2024 for randomized controlled trials (RCTs) comparing the efficacy of Anisodamine in preventing the development of CIN. Outcomes of interest included the incidence of CIN, serum creatinine levels, and estimated glomerular filtration rate (eGFR). A random-effects model was used for pooling standard mean differences (SMDs) and odds ratios (ORs) with a 95% CI. Statistical significance was considered at a *P* less than 0.05.

**Results:**

Three RCTs involving 563 patients were included. Anisodamine was associated with a reduction in the incidence of CIN [OR: 0.44; 95% CI: 0.28, 0.69; *P*=0.0003], a reduction in serum creatinine levels at 48 [SMD: −6.78; 95% CI: −10.54,−3.02; *P*=0.0004] and 72 h [SMD: −6.74; 95% CI: −13.33,−0.15; *P*=0.03], and a higher eGFR at 24 [SMD: 5.77; 95% CI: 0.39, 11.14; *P*=0.03], and 48 h [SMD: 4.70; 95% CI: 2.03,7.38; *P*=0.0006]. The levels of serum creatinine at 24 h and eGFR value at 72 h were comparable between both groups.

**Conclusions:**

Anisodamine has demonstrated clinical efficacy in ameliorating the development of CIN post-PCI in AMI patients. Large, multi-centric RCTs are warranted to evaluate the robustness of these findings.

## Introduction

Highlights

- Contrast-induced nephropathy (CIN) is a common complication of percutaneous coronary intervention (PCI).
- Anisodamine hydrobromide demonstrated clinical efficacy in preventing CIN development.
- Anisodamine treatment is associated with higher estimated glomerular filtration rate and lower serum creatinine values.
- Further large multi-centric trials are needed to evaluate the robustness of these findings.

**Figure:**
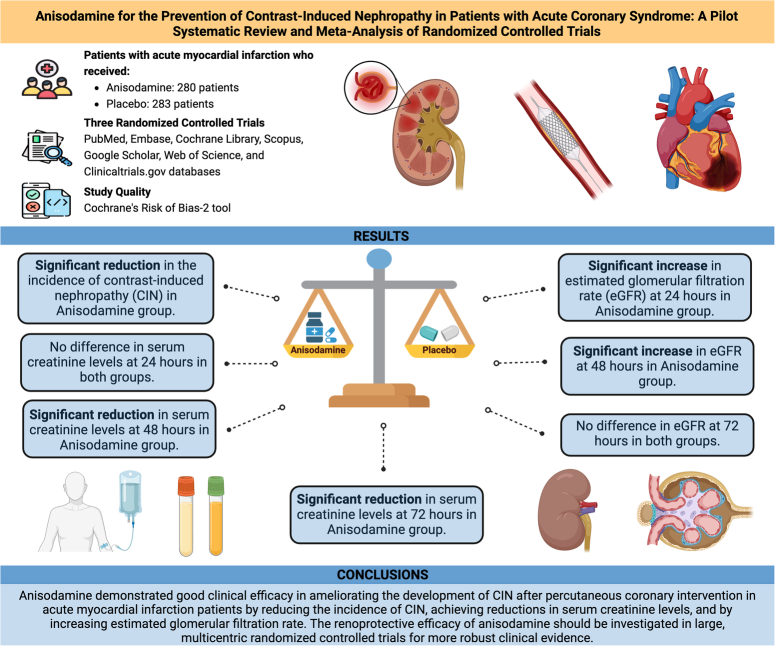


Contrast-induced nephropathy (CIN) is defined as an increase in serum creatinine (Cr) by greater than 25% or greater than or equal to 0.5 mg/dl (44 μmol/l) from baseline within 48 h, excluding other potential causes of nephropathy. Current definitions also support the temporal association of a decline in renal function until seven days post-contrast intake to CIN, if no probable cause of nephropathy exists^1^. CIN ranks as the third most prevalent cause of hospital-acquired acute renal injury or acute kidney injury (AKI), comprising ~12% of all cases, following renal hypoperfusion (42%) and postoperative renal injury (18%)^2^. The incidence ranges from negligible in individuals with normal renal function to as high as 50% in the high-risk populations^3,4^. Factors contributing to a heightened CIN risk include pre-existing renal impairment, diabetes mellitus, advanced age, peri-procedural dehydration, congestive heart failure, diuretic therapy, volume and type of administered contrast, and concurrent use of other nephrotoxic drugs^5^. The development of CIN is associated with prolonged hospital stays, heightened morbidity and mortality, and increased healthcare costs^6,7^. With the rising prevalence of coronary angiography, interventional procedures, and the usage of contrast media, CIN remains a growing concern, particularly in high-risk patients with chronic kidney disease, diabetes, hypertension, and kidney failure.

Unfortunately, no definitive established treatment exists for CIN, emphasizing the importance of prevention in management. Current guidelines advise prophylactic peri-procedural hydration with normal saline, particularly in high-risk patients, to avoid the development of CIN post-procedure^8,9^. Some literature supports sliding-scale intravenous hydration protocol, but the results have been inconsistent^8,10^. The quest for treatment options for CIN has been longing since forever. Previous reports have demonstrated questionable efficacies and equivocal results for acetylcysteine, sodium bicarbonate, dopamine, anti-oxidants, and calcium channel blockers^8,10–15^. Similarly, research into atrial natriuretic peptide, brain natriuretic peptide, and statins has also demonstrated conflicting results for their reno-protective effects^16–21^. This lacuna in the management guidelines for CIN necessitates investigations to explore newer reno-protective molecules and identify optimal strategies for reducing the incidence and mortality associated with CIN.

Anisodamine is a tropane alkaloid derived from Anisodus tanguticus and it shares a structural resemblance with atropine^22^. Like atropine, it exhibits non-specific cholinergic antagonistic activity but is less potent than atropine^23^. Since its first synthesis in 1975, Anisodamine has been therapeutically used for the treatment of shock, improving microcirculation, and respiratory dysfunction, and managing organophosphate poisoning^24–27^. Intracoronary administration of Anisodamine is effective for reversing coronary no-flow phenomenon^28,29^. Numerous models have demonstrated Anisodamine to have reno-protective effects, particularly in glycerol- and lipopolysaccharide-induced AKI^30,31^. Anisodamine has also demonstrated efficacy in ameliorating inflammasome activation in rhabdomyolysis-induced AKI^32^.

In this systematic review and meta-analysis, our objective is to synthesize results from randomized controlled trials (RCTs) to assess the reno-protective effects of Anisodamine in patients with ACS undergoing PCI.

## Methods

This meta-analysis was registered at the PROSPERO Registry for Systematic Reviews and Meta-Analysis (CRD42024498561) and was conducted following Preferred Reporting Items for Systematic Review and Meta-Analysis (PRISMA) guidelines [Supplementary Digital Content 1, http://links.lww.com/MS9/A495]^33,34^. This work has been reported per AMSTAR [Supplementary Digital Content 2, http://links.lww.com/MS9/A496] guidelines.

### Data sources and search strategy

Two investigators (H.J. and R.M.O.) systematically collected data by performing a comprehensive literature search on databases including PubMed, Embase, Cochrane Library, Scopus, Google Scholar, Web of Science, and clinicaltrials.gov from their inception to 15 January 2024 to gather randomized controlled trials (RCTs) assessing the efficacy of anisodamine in preventing the development of CIN in patients with ACS. The search methodology consisted of a combination of the Boolean operators “AND” and “OR” with the following keywords: “anisodamine”, “anisodamine hydrobromide”, and “contrast-induced nephropathy” [Supplementary Table S1, Supplemental Digital Content 3, http://links.lww.com/MS9/A497]. To ensure completeness, the reference list of the included studies was carefully assessed to identify potentially relevant records. Redundant records were removed using the EndNote X7 software (Clarivate Analytics).

### Eligibility criteria

We identified RCTs that met the following inclusion criteria: (a) patients with acute coronary syndromes, (b) had one group that received anisodamine, (c) had a comparator that received a placebo, (d) and evaluated at least one of the outcomes of interest: incidence of CIN, change in serum creatinine, and change in estimated GFR (eGFR). Our analysis excluded studies with insufficient data, reviews, letters, case reports, and editorials. No language restriction was used in identifying the studies.

### Study selection and data extraction

Initially, two investigators (H.J. and R.M.O.) independently assimilated articles per the pre-defined PICO inclusion criteria using title and abstract screening. Those articles that were identified as potentially relevant underwent a full-text review and were subjected to a more thorough evaluation. A third investigator (J.J.) was consulted in case of any discrepancy, or it was resolved with consensus by reaching an agreement between the investigators. The data extracted from each trial included: the surname of the first author, year, sample size, age, number of males and participant-specific data including BMI, left ventricular ejection fraction (LVEF), hypertension (HTN), dyslipidemia, current smoking, and chronic kidney disease (CKD).

### Quality appraisal

Two researchers (R.M.O. and H.J.) independently assessed the quality of the included studies using Cochrane’s Risk of Bias-2 (RoB-2) tool, and to ensure reliability, their results were compared^35^. Each study was categorized as having a low, high, or some concerns of risk of bias for each of these factors. If a study was deemed to have a “high risk” of bias in any one category, it was classified as having an overall “high risk of bias”.

### Data synthesis

All statistical analysis for this meta-analysis was performed using RevMan, version 5.4 (Nordic Cochrane Centre). The results from the included studies were presented as standard mean deviations (SMDs) and odds ratios (ORs) with standard 95% CIs by pooling using the Mantel–Haenzel random-effects model. Forest plots were generated to depict the outcomes visually. In all cases, the statistical significance was considered when the *P* value was below 0.05.

To assess the degree of heterogeneity arising from variability in trial methodologies and study populations, we utilized the Higgins I^2^ metric, with a value of less than 50% indicating low heterogeneity, a value exceeding 50% indicating moderate heterogeneity, and a value greater than 75% indicating significant heterogeneity^36^. Any value of Higgins I^2^ metric greater than 50% warranted a sensitivity analysis using the “leave-one-out” method.

## Results

**Figure 1:**
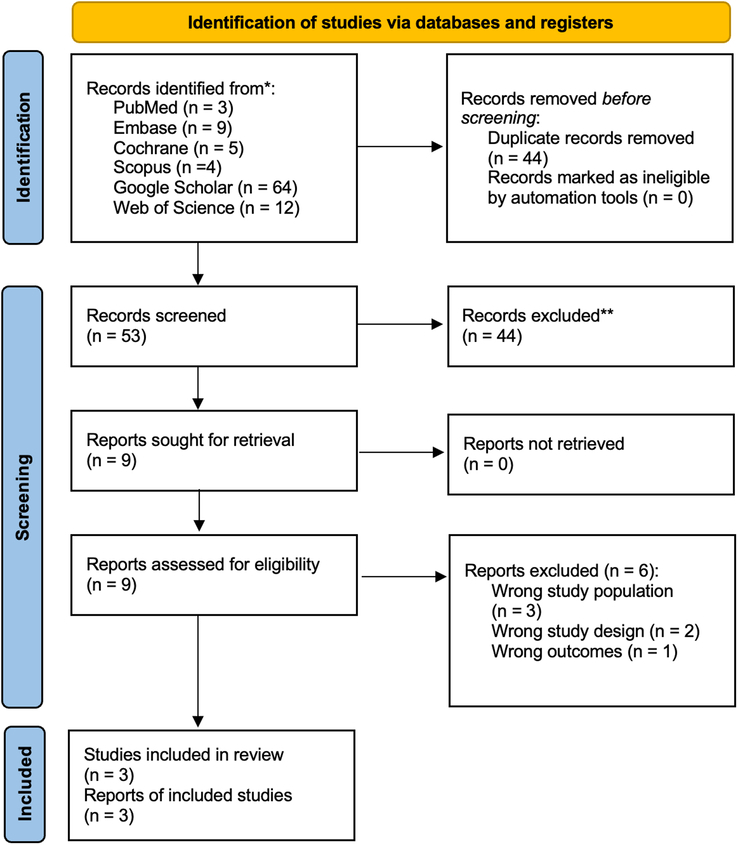
The Preferred Reporting Items for Systematic Reviews and Meta-Analyses (PRISMA) 2020 flowchart.

The search yielded a total of 97 relevant articles. Following the removal of redundant records (*n*=44), a total of 53 articles were subjected to preliminary screening using titles and abstracts. During the preliminary screening, 44 articles were excluded. Subsequently, a total of 9 articles underwent a full-text review, following which 6 were excluded due to various reasons, including wrong study population (*n*=3), wrong study design (*n*=2), and wrong outcomes (*n*=1). Finally, 3 studies were included in the final analysis and were published between 2011 and 2012^37–39^. The PRISMA flowchart outlines the systematic literature search and the study selection process (Fig. 1).

### Study and patient characteristics

The total number of participants was 563. Out of all these patients with acute coronary syndrome, 280 were administered anisodamine, and 283 were administered placebo/normal saline. The baseline characteristics of all included studies are depicted in Table 1.

**Table 1:** Baseline characteristics of included studies

	No. patients; (*n*)		Mean age; mean±SD		No. males; *n* (%)		BMI; mean±SD		LVEF; %		HTN; *n* (%)		Dyslipidemia; *n* (%)		Current Smoking; *n* (%)		CKD; *n* (%)	
Author name (year)	I	C	I	C	I	C	I	C	I	C	I	C	I	C	I	C	I	C
Geng *et al*. (2012)^37^	132	128	65.1±6.9	63.8±6.2	100 (75.7)	90 (70.3)	25.2±2.8	24.8±3.6	63.6±6.9	62.4±7.6	82 (63.6)	70 (54.7)	74 (57.4)	67 (52.3)	78 (59.1)	66 (51.6)	NA	NA
Wang *et al*. (2011)^38^	60	66	56.9±11.2	54.9±12.3	51 (85)	57 (86.3)	25.1±3.1	25.2 ±3.2	60.3±9.5	58.4±10.8	36 (60)	34 (51.5)	15 (25)	13 (19.7)	36 (60)	31 (47)	1 (1.7)	2 (3.0)
Wang *et al*. (2012)^39^	88	89	55±13.2	54.5±12.73	79 (89.8)	79 (88.8)	25.4±3.6	25.5±3.2	58.3±9.2	56.7±10.8	39 (44.3)	44 (49.4)	51 (58)	49 (55.1)	53 (60.2)	56 (62.9)	5 (5.7)	4 (4.5)

C, control (placebo); CKD, chronic kidney disease; HTN, hypertension; I, intervention (Anisodamine); LVEF, left ventricular ejection fraction; NA, not applicable.

### Outcomes

#### Incidence of CIN

The incidence of CIN was reported in three trials^37–39^. Treatment with anisodamine demonstrated a significant reduction in the incidence of CIN [OR: 0.44; 95% CI: 0.28, 0.69; *P*=0.0003; I^2^=0%] (Supplementary Figure S1, Supplemental Digital Content 3, http://links.lww.com/MS9/A497). Multivariable logistic regression for the association between anisodamine and CIN illustrated a significant relation, with anisodamine treatment showing a lower association with CIN than placebo [OR: 0.33; 95% CI: 0.19, 0.62; *P*=0.0005; I^2^=0%] (Supplementary Figure S2, Supplemental Digital Content 3, http://links.lww.com/MS9/A497).

#### Serum creatinine at 24 h

The serum creatinine levels at 24 h were reported in two trials^37,38^. Treatment with anisodamine did not demonstrate a significant reduction in serum creatinine at 24 h after PCI [SMD: −3.94; 95% CI: −8.26, 0.39; *P*=0.07; I^2^=19%] (Supplementary Figure S3, Supplemental Digital Content 3, http://links.lww.com/MS9/A497).

#### Serum creatinine at 48 h

The serum creatinine levels at 48 h were reported in three trials^37–39^. Treatment with anisodamine demonstrated a significant reduction in serum creatinine at 48 h after PCI [SMD: −6.78; 95% CI: −10.54, −3.02; *P*=0.0004; I^2^=0%] (Supplementary Figure S4, Supplemental Digital Content 3, http://links.lww.com/MS9/A497).

#### Serum creatinine at 72 h

The serum creatinine levels at 72 h were reported in three trials^37–39^. Treatment with anisodamine demonstrated a significant reduction in serum creatinine at 72 h after PCI [SMD: −6.74; 95% CI: −13.33, −0.15; *P*=0.03; I^2^=72%] (Supplementary Figure S5, Supplemental Digital Content 3, http://links.lww.com/MS9/A497). Considering moderate heterogeneity, omitting Geng 2012 dropped the I^2^ to 0%.

#### eGFR at 24 h

The eGFR at 24 h was reported in three trials^37–39^. Treatment with anisodamine demonstrated a significant increase in eGFR at 24 h after PCI [SMD: 5.77; 95% CI: 0.39, 11.14, *P*=0.03; I^2^=71%] (Supplemental Figure S6, Supplemental Digital Content 3, http://links.lww.com/MS9/A497). Considering moderate heterogeneity, omitting Geng 2012 dropped the I^2^ to 0%.

#### eGFR at 48 h

The eGFR at 48 h was reported in three trials^37–39^. Treatment with anisodamine demonstrated a significant increase in eGFR at 48 h after PCI [SMD: 4.70; 95% CI: 2.03, 7.38; *P*=0.0006; I2=31%] (Supplementary Figure S7, Supplemental Digital Content 3, http://links.lww.com/MS9/A497).

#### eGFR at 72 h

The eGFR at 72 h was reported in three trials^37–39^. Treatment with anisodamine did not demonstrate any significant increase in eGFR at 72 h after PCI [SMD: 5.60; 95% CI: −0.23, 11.43; *P*=0.06; I2=78%] (Supplementary Figure S8, Supplemental Digital Content 3, http://links.lww.com/MS9/A497). Considering severe heterogeneity, omitting Geng 2012 dropped the I^2^ to 0%.

### Quality assessment

The quality appraisal for the included trials was conducted using Cochrane’s RoB-2 tool, and the results are depicted in Supplementary Figure S9, Supplemental Digital Content 3, http://links.lww.com/MS9/A497, S10, Supplemental Digital Content 3, http://links.lww.com/MS9/A497. No risk of bias was noted in all the included trials.

## Discussion

To the best of our knowledge, this is the first meta-analysis to date that evaluates the efficacy and reno-protective effects of anisodamine in CIN in patients with ACS. Our meta-analysis reported a decrease in the incidence of CIN, a reduction in the level of serum creatinine at 48 h and 72 h, and an increase in eGFR at 24 h, and 48 h. Multivariate logistic regression result also demonstrates a lower association of CIN with anisodamine as compared to placebo.

Due to an increase in the prevalence of coronary artery disease in the past few decades, the incidence of elective and non-elective PCI procedures has been reported to be increasing widely^40^. Due to the rising procedure rates, the risk of CIN occurrence is also increasing^41^. Individuals who develop CIN post-PCI demonstrate an increased risk for short- and long-term mortality as compared to the general population^42^. CIN frequently complicates post-PCI duration leading to increased mortality, morbidity and longer hospitalization^43^.

Anisodamine has demonstrated vasodilating effects through ɑ-1 adrenergic blockade at high concentrations, relieving vasospasms and improving microcirculation^44^. The mechanism behind its clinical efficacy on microcirculation has been postulated to be the inhibition of platelet aggregation via thromboxane inhibition^26^. This clinical benefit has been explored in nephropathies in recent practice and is largely observed due to the improvements in renal microcirculation^22,30^. The exact mechanism behind the development of CIN is yet to be elucidated. However, the most important mechanisms proposed are—direct oxidative damage, renal medullary hypoxia, and apoptosis^45,46^. In our analysis, we reported improved clearance and glomerular function, possibly attributable to the prostaglandin-increasing effects of anisodamine, leading to enhanced renal perfusion^26^. Anisodamine also leads to an increase in glomerular blood flow by about 15–40% depending on its dosage, which counteracts renal ischemia to ameliorate the damage to renal microvasculature and renal tissues^47^. The reno-protective effects may also stem from anisodamine’s antioxidant properties, countering contrast-induced renal damage mediated by free radical damage from contrast media^48^. Anisodamine also leads to the activation of superoxide dismutase, an enzyme that scavenges reactive oxygen species and protects renal tissues from free radicals ensued damage produced by the administration of iodinated contrasts^49^. Nevertheless, most of the current pathophysiological evidence comes from animal models that investigated the mechanism behind the observation where pretreatment with Anisodamine ameliorates renal dysfunction, and the exact mechanism needs further investigation^31^. Another possible explanation is the lowering of Ca2+ load in cases of acute ischemic renal failure originating from upstream microcirculature^50^. Alternatively, it might involve the inhibition of apoptosis and the suppression of inflammatory cytokine production^51^. Though supported by evidence, most of the above-listed mechanisms need further investigation.

### Limitations

This meta-analysis has several limitations that must be addressed before interpreting the findings. Firstly, the small number of studies, and by extrapolation, the total sample size may affect the generalizability of the results. Secondly, this pooled analysis is of individual studies and not of individual patient data hence individual patient characteristics could not be considered. Thirdly, for some outcomes, high heterogeneity was reported for some outcomes, which tried to address by sensitivity analysis using the “leave-one-out” approach. However, this could introduce variability and affect the reliability of the effect estimate for some outcomes. Fourthly, all trials that were included in this meta-analysis were conducted in China, which attention when extrapolating the results to other population cohorts. Lastly, all the trials included in this meta-analysis had statistically significant differences in the number of male versus female patients enrolled, with males having over 70% representation. Hence, the extrapolation of the results of this study to female sex could introduce bias in outcomes.

## Conclusion

In conclusion, CIN has been demonstrated to be an increasingly prevalent condition post-PCI and is associated with poor outcomes. Anisodamine hydrobromide is an alkaloid with proven reno-protective effects in patients undergoing PCI for AMI, leading to decreased CIN incidence. Pairing peri-procedural hydration with anisodamine should be evaluated in large, multi-center RCTs for more robust evidence against anisodamine reno-protective effects.

## Ethical approval

Ethical approval was not required for this systematic review.

## Consent

Informed consent was not required for this systematic review.

## Source of funding

Not applicable.

## Author contribution

H.J.: conceptualization, methodology, writing—original draft, writing—review and editing, supervision, project administration; R.M.O.: formal analysis, methodology, writing—original draft, writing—review and editing, supervision; J.J.: validation, investigation, writing—original draft, writing—review and editing; D.D.: data curation, writing—original draft, writing—review and editing; A.M.H.: Visualization, writing—original draft, writing—review and editing; M.D.M.M.: writing—original draft, writing—review and editing; H.S.: writing—original draft, writing—review and editing; A.M.: writing—original draft, writing—review and editing; H.Y.: writing—original draft, writing—review and editing; S.P.: writing—original draft, writing—review and editing; R.Y.: writing—original draft, writing—review and editing.

## Conflicts of interest disclosure

The authors declare no conflicts of interest.

## Research registration unique identifying number (UIN)

PROSPERO Registration ID: CRD42024498561.

## Guarantor

Hritvik Jain.

## Data availability statement

Not applicable.

## Provenance and peer review

Not applicable.

## Supplementary Material

SUPPLEMENTARY MATERIAL
