# Supplementary material for: Anisodamine for the prevention of contrast-induced nephropathy in patients with acute coronary syndrome: a pilot systematic review and meta-analysis of randomized controlled trials
Source: Ann Med Surg (Lond). 2024 May 21;86(7):4123–9. doi: 10.1097/MS9.0000000000002181 (PMC11230748; doi:10.1097/MS9.0000000000002181)

**Supplementary Material**

**Anisodamine for the Prevention of Contrast-Induced Nephropathy in Patients with Acute Coronary Syndrome: A Pilot Systematic Review and Meta-Analysis of Randomized Controlled Trials**

**Supplementary Table S1**. Search strategies for online databases.

**Supplementary Figure S1**. Forest plot for incidence of CIN.

**Supplementary Figure S2**. Forest plot for the association between CIN and AIN.

**Supplementary Figure S3**. Forest plot for serum creatinine levels at 24 hours.

**Supplementary Figure S4**. Forest plot for serum creatinine levels at 48 hours.

**Supplementary Figure S5**. Forest plot for serum creatinine levels at 72 hours.

**Supplementary Figure S6**. Forest plot for eGFR values at 24 hours.

**Supplementary Figure S7**. Forest plot for eGFR values at 48 hours.

**Supplementary Figure S8**. Forest plot for eGFR values at 72 hours.

**Supplementary Figure S9**. Risk of bias assessment using Cochrane’s RoB-2 tool.

**Supplementary Figure S10**. Cochrane’s RoB-2 tool summary plot.

This supplemental material has been provided by the authors to give readers additional information about their work.

**Supplementary Table S1**. Search strategies for online databases

| Search Number | Database | Search Strategy | Number of Results |
| --- | --- | --- | --- |
| #1 | PubMed | ("anisodamine"[Supplementary Concept] OR "anisodamine" OR "anisodamine hydrobromide" OR "racanisodamine" OR "6-hydroxyhyoscyamine") AND ("contrast-induced nephropathy" OR "contrast induced nephropathy") | 3 |
| #2 | Embase | (“anisodamine” OR “anisodamine hydrobromide” OR “racanisodamine” OR “6-hydroxyhyoscamine”) AND (“contrast-induced nephropathy” OR “contrast induced nephropathy”) | 9 |
| #3 | Cochrane | (“anisodamine” OR “racanisodamine”) AND (“contrast-induced nephropathy” OR “contrast induced nephropathy”) | 5 |
| #4 | Scopus | (TITLE-ABS-KEY(anisodamine) OR TITLE-ABS-KEY(racanisodamine)) AND (TITLE-KEY-ABS(contrast-induced nephropathy) OR TITLE-ABS-KEY(contrast induced nephropathy)) | 4 |
| #5 | Google Scholar | (“anisodamine” OR “anisodamine hydrobromide” OR “racanisodamine” OR “6-hydroxyhyoscamine”) AND (“contrast-induced nephropathy” OR “contrast induced nephropathy”) | 64 |
| #6 | Web of Science | (“anisodamine” OR “anisodamine hydrobromide” OR “racanisodamine” OR “6-hydroxyhyoscamine”) AND (“contrast-induced nephropathy” OR “contrast induced nephropathy”) | 12 |

**Supplementary Figure S1**. Forest plot for incidence of CIN.


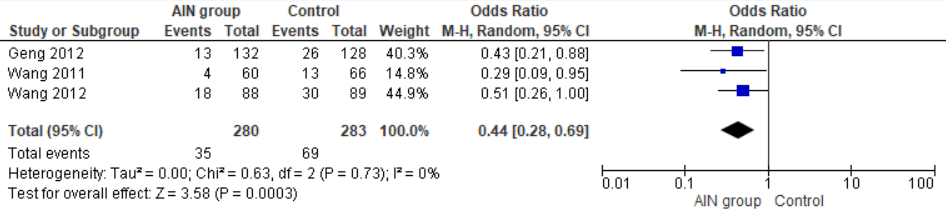


**Supplementary Figure S2**. Forest plot for the association between CIN and AIN.


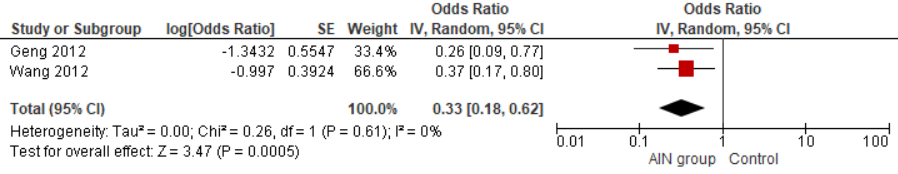


**Supplementary Figure S3**. Forest plot for serum creatinine levels at 24 hours.


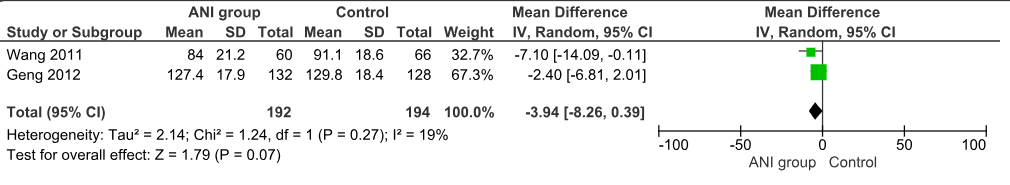


**Supplementary Figure S4**. Forest plot for serum creatinine levels at 48 hours.


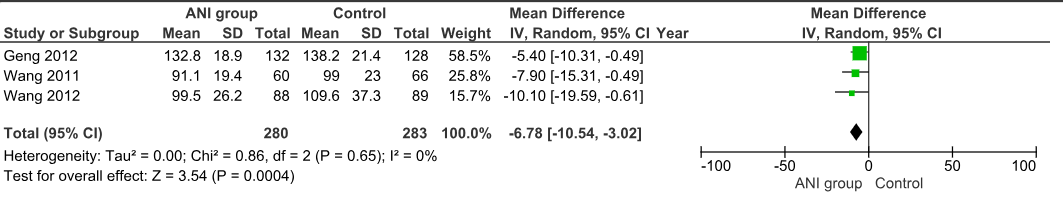


**Supplementary Figure S5**. Forest plot for serum creatinine levels at 72 hours.


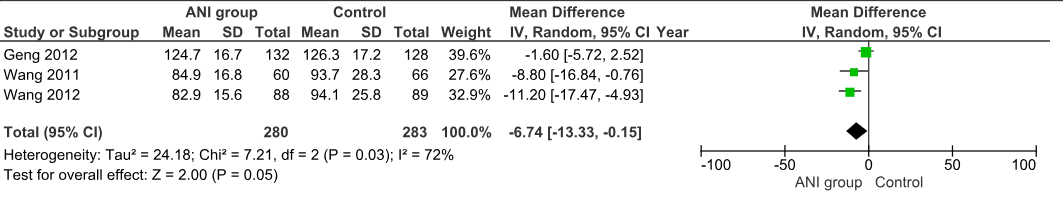


**Supplementary Figure S6**. Forest plot for eGFR values at 24 hours.


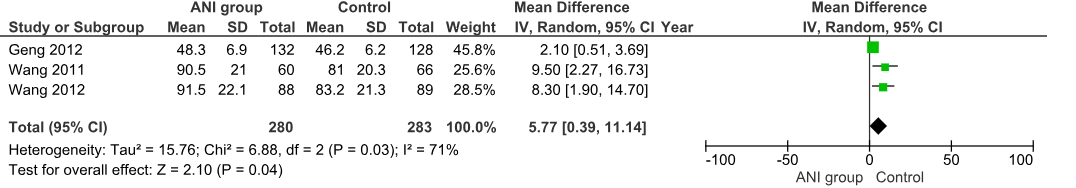


**Supplementary Figure S7**. Forest plot for eGFR values at 48 hours.


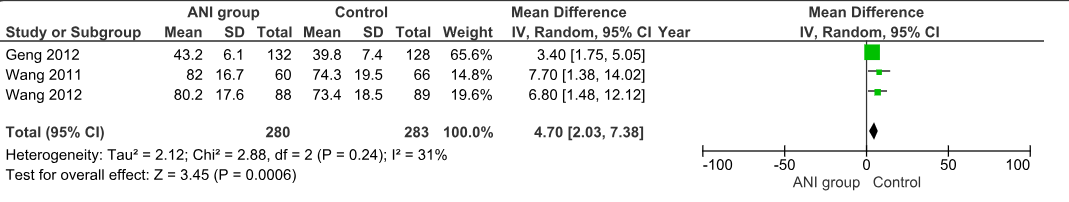


**Supplementary Figure S8**. Forest plot for eGFR values at 72 hours.


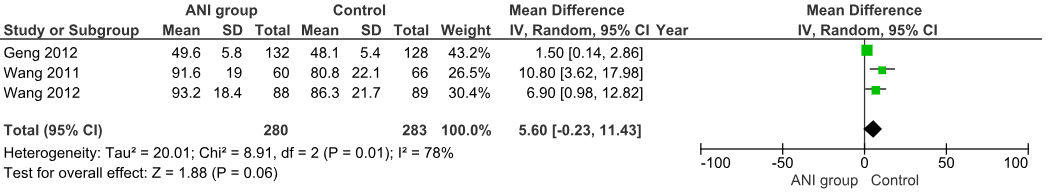


**Supplementary Figure S9**. Risk of bias assessment using Cochrane’s RoB-2 tool.


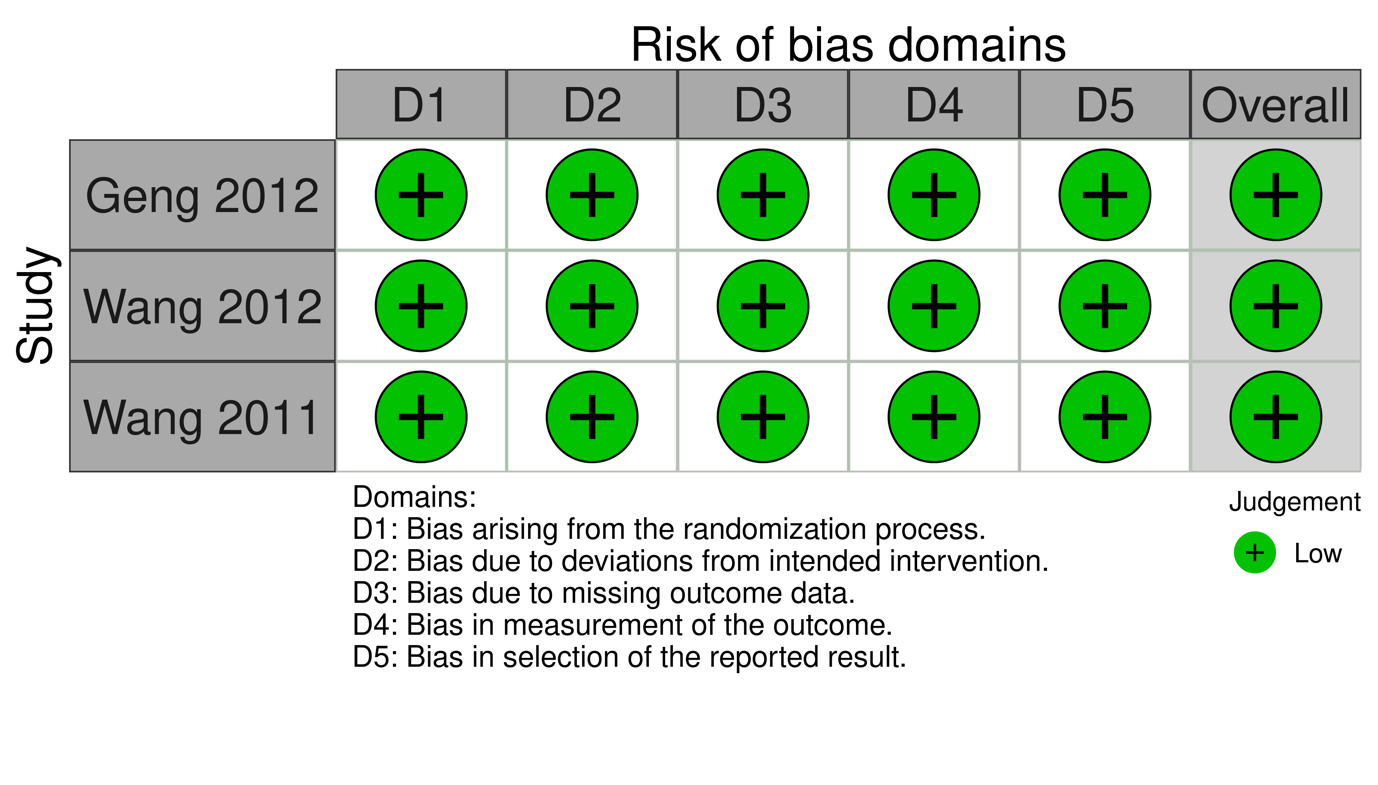


**Supplementary Figure S10**. Cochrane’s RoB-2 tool summary plot.


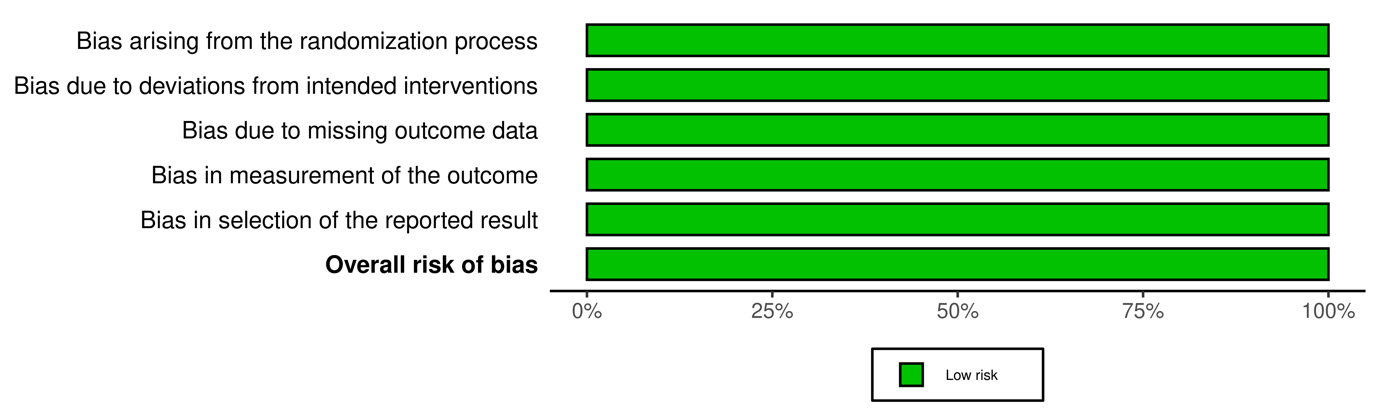

Supplement: SUPPLEMENTARY MATERIAL [file ms9-86-4123-s003.docx]
